# Weak interactions in furan dimers

**DOI:** 10.1007/s10822-018-0163-5

**Published:** 2018-09-14

**Authors:** Irena Majerz

**Affiliations:** Faculty of Pharmacy, Wroclaw Medical University, Borowska 211a, 50-556 Wrocław, Poland

**Keywords:** Furan, 2,3-Dihydrofuran, 2,5-Dihydrofuran, Tetrahydrofuran, QTAIM, NCI, Hydrogen bond, Stacking

## Abstract

Dimers of furan, 2,3-dihydrofuran, 2,5-dihydrofuran and tetrahydrofuran were investigated with the use of theoretical methods to determine the interactions that keep the molecules together. The QTAIM and NCI methods confirmed that for furan dimers the C–H⋯O hydrogen bond and stacking interactions can form the dimers with similar energy. For 2,3-dihydrofuran, 2,5-dihydrofuran and tetrahydrofuran, the decisive mechanism of dimer formation is the stacking interaction between the furan rings.

**Electronic supplementary material:**

The online version of this article (10.1007/s10822-018-0163-5) contains supplementary material, which is available to authorized users.

## Introduction

For a long time furan has been attracting more and more attention because of its wide occurrence in biomolecules, macromolecules and drugs. Furan derivatives are known for their pharmacological activities: cytotoxic and antitumor properties [1, 2], antispasmodic [3] and anti-feeding [4]. Another reason for investigating furan is its potential technological application. Polyfuran is a conducting polymer with relatively high electric conductivity [5, 6]. Furan derivatives reveal high optical nonlinearity and can be used to design optical and electroluminescent devices [7, 8]. Furan compounds can be applied for dye-sensitized solar cells [9] and organic dyes containing a furan moiety for high-performance dye sensitized solar cells [10]. Furan importance is associated with many potential applications in material science, natural product synthesis and other fields of chemistry. Moreover, furan is a very common compound existing in food. Due to furan low evaporation temperature, it is also one of the most popular air impurities.

Except for the practical reasons listed above, furan is popular in the literature because it is an aromatic heteroatomic compound [11–13], and the aromatic ring with a delocalized electron cloud and a heteroatom included into the ring determines the furan intermolecular interaction as well as the interaction of furan with other molecules. Therefore, furan and its complexes with small molecules were the subject of many experimental and theoretical studies [14–18].

The molecular structure of furan was investigated with many experimental methods: microwave spectroscopy [19, 20], electron diffraction and rotational spectroscopy [21], X-ray diffraction [22, 23]. All the experimental methods revealed that the furan ring is planar with C_2v_ symmetry. The experimental molecular structure was confirmed by theoretical calculations [13, 18, 23–25].

The supramolecular structure of furan in the gaseous and liquid phases is connected with the formation of molecular clusters [23, 24] and the simplest cluster of furan is a dimer. Both furan molecules are linked together with a hydrogen bond or a weak van der Waals interaction. The furan dimers were investigated previously [24] at B3LYP/6-311G(d) level of theory. The authors found four furan dimer isomers. The lowest energy dimer was a cyclic structure bound by two CH⋯O hydrogen bonds and the cooperativity of these bonds was found as a force stabilizing the structure. The second conformer with a relative energy of 0.76 kcal/mol was linked by a single CH⋯O hydrogen bond. In other conformers hydrogen atoms belonging to the furan molecule were directed to the carbons or hydrogens of the second molecule participating in the dimer. The solid state structure of furan in which the molecules are linked by the interaction of the hydrogen atom of one molecule and the oxygen and carbon atoms of the neighboring molecule was reproduced in the DFT calculation for a single unit cell with periodic boundary conditions [25].

**Scheme 1:**
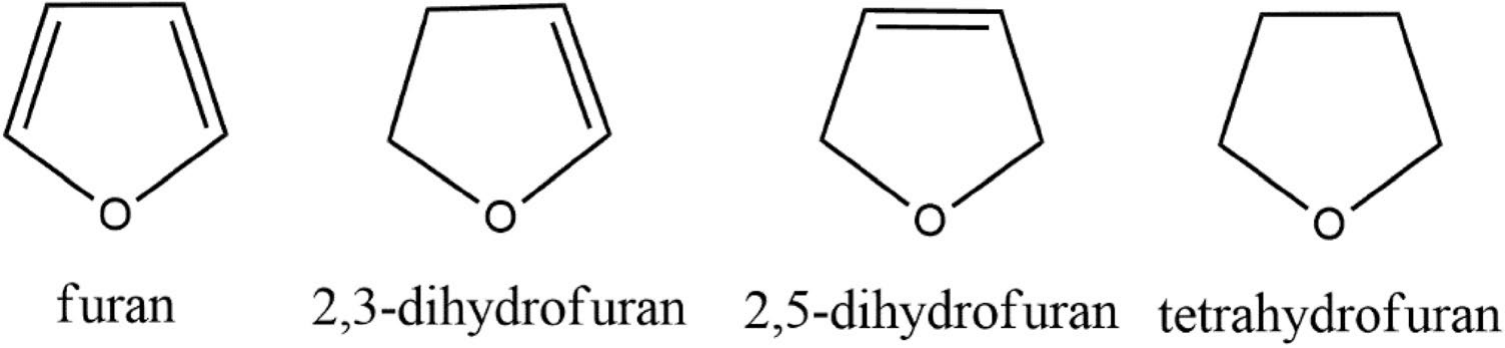
Monomers of the investigated furan derivatives

The main part of the furan dimer investigation is an explanation which interaction is responsible for linking the molecules together. Quantum theory of atom in molecules (QTAIM), noncovalent interaction (NCI) approaches and energy decomposition cast light on the interaction of the furan derivatives studied in this paper. Monomers that form the investigated dimers are presented in Scheme 1.

## Computational details

The optimization of the investigated molecules was performed with gaussian09 package [26] at B3LYP-GD3/6-311++G**, B3LYP-GD3/aug-cc-pVTZ with Grimme dispersion [27] and M062X/6-311++G** level of theory. To determine the energies of the investigated dimers precisely, both dispersion correction and basis superposition errors (BSSE) using the standard counterpoise (CP) method [28] was used [29]. Zero point energy correction in the energy calculation for all the investigated compounds was included.

The conformers of the investigated dimers obtained using the calculation methods listed above are collected in Table 1. Independent of the calculation method the structures of the dimers characterized by the angles between the furan planes are analogous. B3LYP-GD3/aug-cc-pVTZ method gave as a result the dimers of tetrahydrofuran and 2,5-dihydrofuran with this same energy but different structure. Among the results obtained with M062X/6-311++G** few structures were characterized with extremely low energy. The results obtained using B3LYP-GD3/6-311++G** method seem to be the best and were used to investigate the interactions linking the molecules in the dimers.

**Table 1:** Comparison of the relative, BSSE- and zero point energy corrected energies and angle between the furan ring planes

B3LYP-GD3/6-311++G**			B3LYP-GD3/aug-cc-pVTZ			M062X/6-311++G**		
Relative energy [kcal/mol]	Angle between ring planes [°]	Distance between ring center [Å]	Relative energy [kcal/mol]	Angle between ring planes [°]	Distance between ring center [Å]	Relative energy [kcal/mol]	Angle between ring planes [°]	Distance between ring center [Å]
Furan								
0.00	1.94	4.4922	0.09	8.53	4.4922	0.51	8.53	4.4922
0.10	86.12	4.4339	0.00	72.61	4.6070	0.49	78.41	4.5136
0.48	60.33	4.4661	0.33	61.15	4.4571	0.09	61.04	4.4408
0.61	6.45	3.8164	0.42	4.11	3.7812	0.06	2.56	3.5509
0.71	17.03	3.7523	0.51	10.04	3.4435	0.00	10.04	3.4435
2,3-Dihydrofuran								
0.00	0.04	3.7953	0.00	0.05	3.7790	0.00	0.04	3.7909
0.03	19.66	3.3710	0.11	17.55	3.7823	0.31	23.24	3.7193
0.80	88.57	3.3496	0.88	88.80	4.3307	0.32	88.80	4.3307
1.19	31.81	4.1143	1.36	31.81	4.1143	1.62	18.95	5.2104
1.30	61.44	4.3601	1.24	60.12	4.3641	1.45	52.99	4.2203
1.64	87.78	4.4961	1.55	89.92	4.4890	1.63	89.92	4.3854
2,5-Dihydrofuran								
0.00	18.95	3.6617	0.00	30.69	4.0265	0.00	9.85	3.4443
0.77	89.95	4.4380	0.68	89.95	4.5361	0.70	89.97	4.4585
0.77	90.00	4.4244	0.68	90.00	4.4163	2.07	89.99	4.3390
0.82	69.72	4.4521	0.75	68.58	4.4320	0.78	69.67	4.4520
1.11	17.38	4.4755	1.06	33.53	4.4688	1.59	33.53	4.4755
1.22	27.37	4.4265	0.00	27.37	4.4224	− 140.77	27.37	4.3001
Tetrahydrofuran								
0.00	39.02	4.0700	0.00	37.38	4.0714	0.33	49.76	3.9108
0.06	9.06	3.8519	0.00	9.19	3.8623	0.00	14.97	3.6543
0.10	28.51	4.0333	0.00	11.65	4.0021	1.54	22.40	4.4827
0.14	11.81	4.0140	0.05	12.62	4.0075	0.38	16.54	3.9029
0.33	87.99	4.5105	1.58	85.37	4.6766	2.21	89.83	4.3684
1.29	80.37	4.6566	1.16	80.37	4.6566	1.73	80.37	4.6566
1.64	35.59	4.3152	1.05	35.59	4.8057	0.84	35.24	4.3152
1.89	32.60	3.8292	0.08	17.66	3.8135	− 150.07	9.99	3.7350

All the optimized equilibrium isomers without imaginary frequencies correspond to the minima on the potential energy surface

In order to investigate the intermolecular interaction linking the furan molecules in the dimers, the QTAIM method was used [30]. The wave functions evaluated for the optimized molecules were used as an input to the AIMALL program [31]. The NCI analysis [32] was performed with the NCI program [33]. The energy decomposition procedure according to Morokuma was performed for the optimized structures [34, 35].

## Results and discussion

### Structure of the investigated dimers

**Fig. 1:**
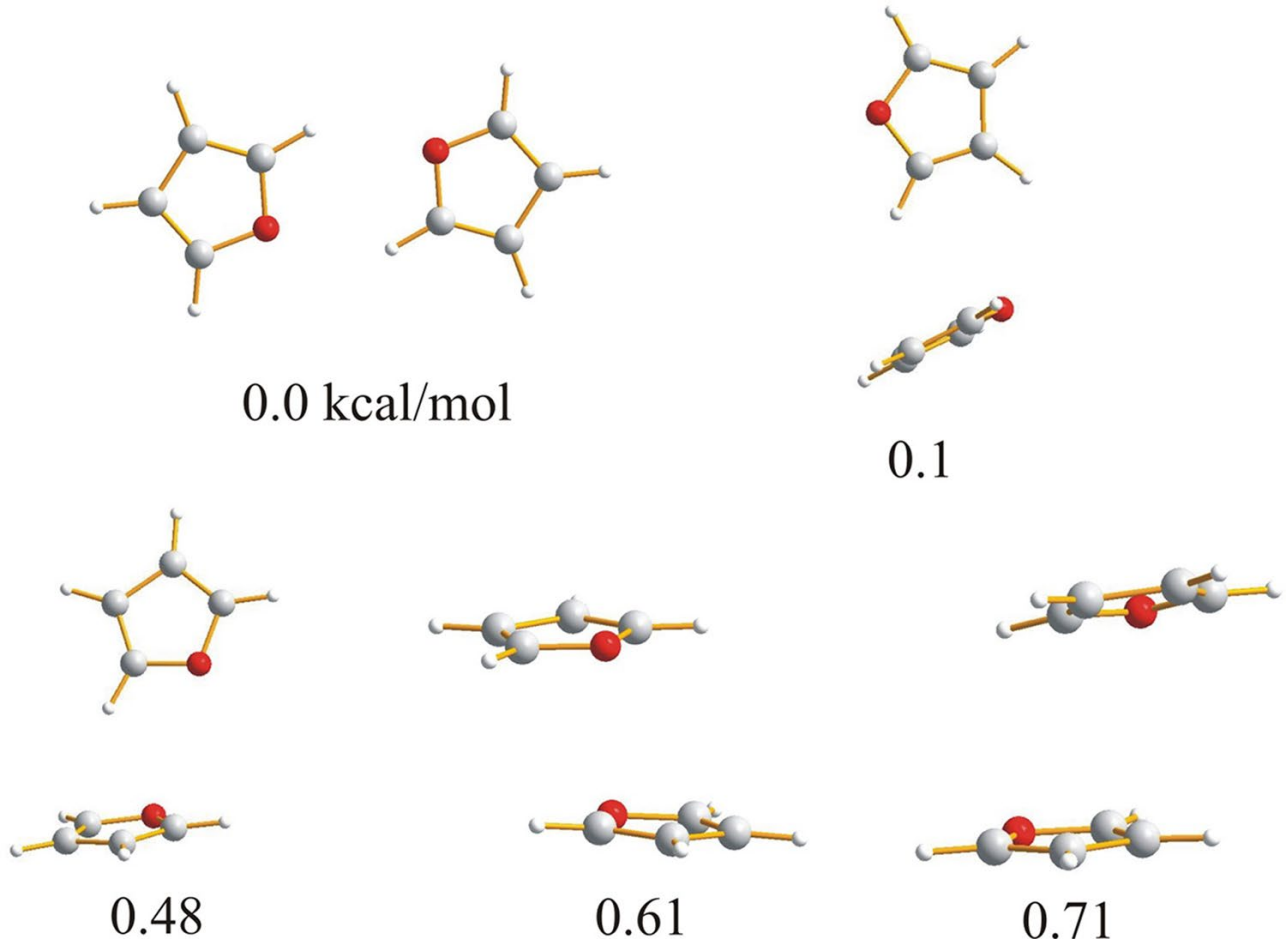
Geometries and relative energies of furan dimers optimized with B3LYP-GD3/6-311++G** method with including BSSE and zero point energy correction. Relative energies in kcal/mol

**Fig. 2:**
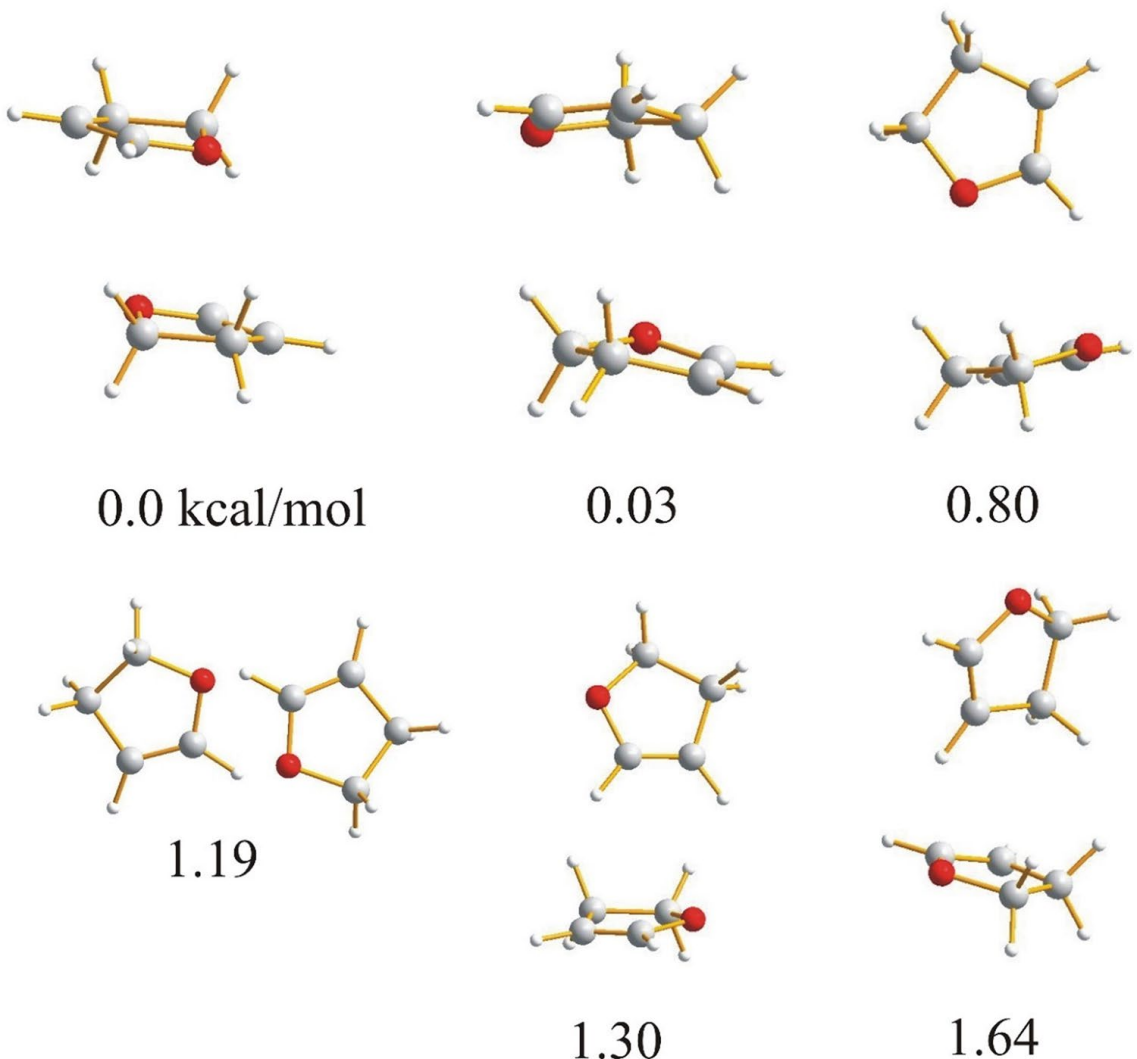
Geometries and relative energies of 2,3-dihydrofuran dimers optimized with B3LYP-GD3/6-311++G** method with including BSSE and zero point energy correction. Relative energies in kcal/mol

**Fig. 3:**
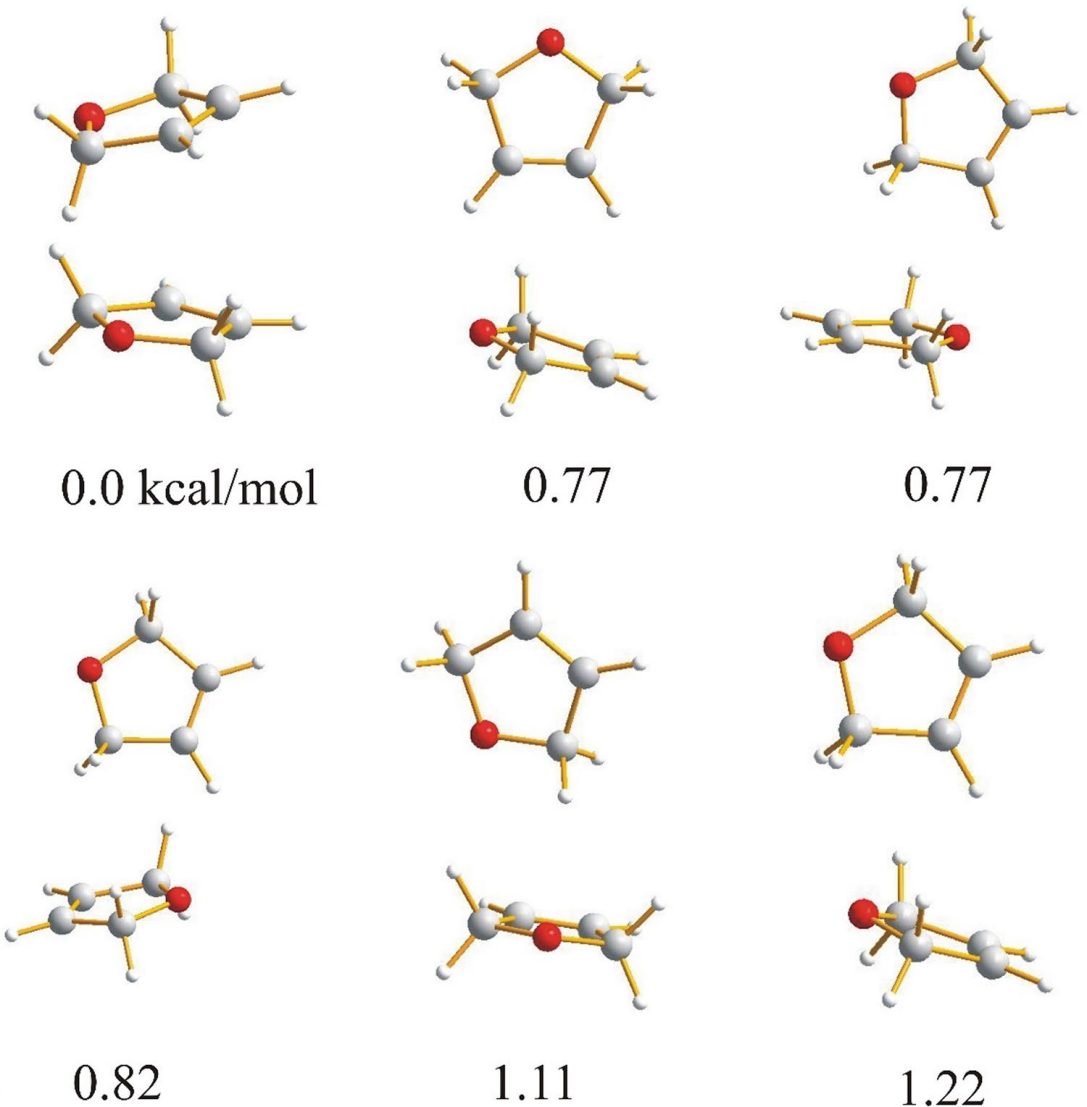
Geometries and relative energies of 2,5-dihydrofuran dimers optimized with B3LYP-GD3/6-311++G** method with including BSSE and zero point energy correction. Relative energies in kcal/mol

**Fig. 4:**
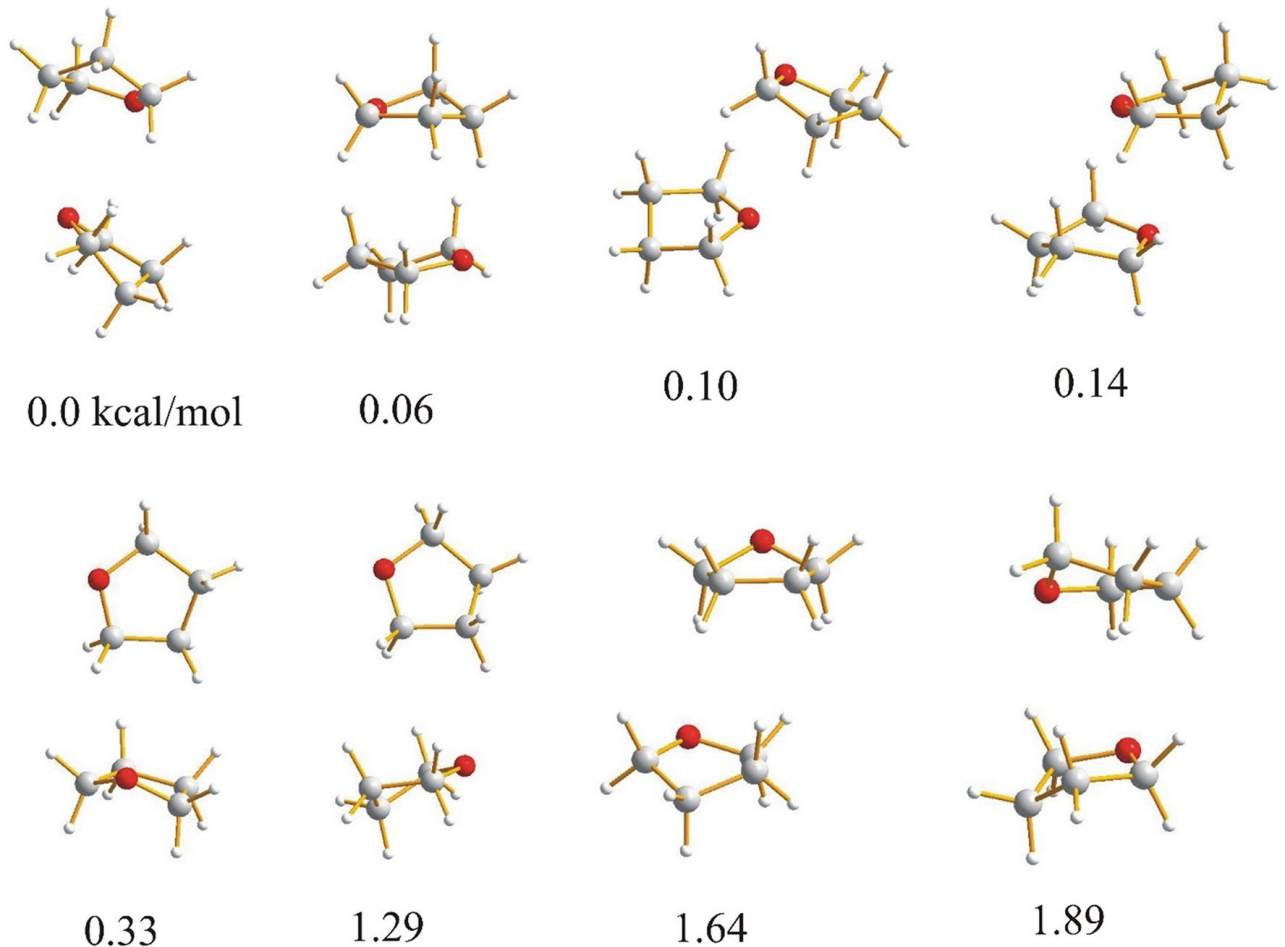
Geometries and relative energies of tetrahydrofuran dimers optimized with B3LYP-GD3/6-311++G** method with including zero point energy correction. Relative energies in kcal/mol

Because the molecules investigated in this paper: furan, 2,3-dihydrofuran, 2,5-dihydrofuran contain an oxygen heteroatom with lone pairs and aromatic electrons, it can be expected that the molecules in the dimer can be linked by weak C–H⋯O or C–H⋯π intermolecular hydrogen bonds or by van der Waals stacking interactions. For tetrahydrofuran, which is not aromatic, the weak C–H⋯O hydrogen bond seems to be the natural intermolecular force linking the molecules together. The optimized dimers of the investigated compounds with the relative energies are presented in Figs. 1, 2, 3 and 4.

The comparison of the results presented in this manuscript with these obtained before [24] demonstrates that the dispersion correction included in our calculation gives a different set of the optimized structures. Five low energy dimers of furan have been found. The lowest energy conformer with two C–H⋯O hydrogen bonds stabilizing the cyclic structure is similar to that found previously [24] but the H⋯O distance is significantly shortened up to 2.4484 Å (C⋯O 3.3510 Å OHO 140.586°). Other furan dimers presented in Fig. 1 are linked by weak interactions. In the conformers with the energy of 0.10 and 0.48 kcal/mol furan protons are directed to the aromatic ring of another furan molecule. The conformers with the energy of 0.61 and 0.71 kcal/mol are characterized by a parallel location of the furan molecules.

For the lowest energy dimers of 2,3-dihydrofuran (Fig. 2) a parallel arrangement of the molecules is typical. The cyclic structure with two nonequivalent CH⋯O hydrogen bonds with the H⋯O distances of 1.6400 Å (C⋯O 2.4406 Å, 126.272°) and 1.5014 Å (C⋯O 2.3008 Å, 125.201°) is characterized by a relative energy of 1.19 kcal/mol. Other dimers with similar energy have a T-shaped location of the molecules. In the dimers of 2,5-dihydrofuran (Fig. 3) and tetrahydrofuran (Fig. 4), the lowest energy structure is characterized by a parallel location of the molecules. For furan and 2,3-dihydrofuran the cyclic structure linking the monomers is one of the lowest energy structures but the linear structure with one C–H⋯O hydrogen bond suggested previously as one of the low energy structures of furan [24] has not been found.

It is characteristic that for furan the cyclic structure is the lowest energy one. For 2,3-dihydrofuran the cyclic structure is characterized by the energy of 1.19 kcal/mol and for 4,5-dihydrofuran and tetrahydrofuran the cyclic structure with two CHO hydrogen bonds has not been found. Formation of the cyclic structure is not possible for the aliphatic CH_2_ group linked to the furan oxygen as the CH_2_ protons cannot be directed to the oxygen electron pairs. In the case of 2,3-dihydrofuran formation of the cyclic dimer is difficult what is expressed by the higher energy.

### Intermolecular interactions in the dimers of furan, 2,3-dihydrofuran, 2,5-dihydrofuran and tetrahydrofuran

Although the geometry of the dimers of the investigated compounds can suggest the type of interactions binding the molecules together, a detailed investigation of the intermolecular interaction must be performed to answer the question which type of interaction is responsible for the dimer formation.

As the starting point for the interaction analysis, the molecular electrostatic potential for all the dimers has been calculated. The electrostatic potential can be obtained by mapping the potentials created in the space around a molecule by its nuclei and electrons on the total electron density. The colours used in the electrostatic potential picture indicate positive and negative charges around the molecule which provides useful information for understanding the shape, size, charge density, delocalization and site of chemical reactivity of the molecules. The electrostatic potential for the dimers presented in Figs. 1, 2, 3 and 4 is depicted in Figs. S1, S2, S3 and S4. The characteristic feature is that the positive charge is located at the hydrogen atoms and the negative is mainly placed at the oxygens. The center of the aromatic ring is negatively charged for furan so the protons can be linked to the oxygen or to the π electron cloud of the second molecule of the dimer. For 2,3- and 2,5-dihydrofuran the negative charge is connected with oxygen and partially with the double bond of the furan ring. The charge of the ring is neutral for tetrahydrofuran. The parallel arrangement of the molecules in dimers is in agreement with the electrostatic interaction and its characteristic feature is that the location of the oxygens of the interacting molecules one under another is impossible.

Both the dimer geometry and the electrostatic potential can suggest the interaction in the investigated dimers, but in the case of very weak interactions additional investigation is necessary. Among the possible interaction between furan molecules, the strongest seems to be the C–H⋯O hydrogen bond with the proton directed at the oxygen lone pair. Taking into account the geometry of the dimers presented in Fig. 1, it is difficult to embed when the dimer is formed by the interaction of the proton with the lone pair of the furan oxygen or may be by the interaction of the proton with the π electron cloud of the aromatic ring. Except for the C–H⋯O and C–H⋯π hydrogen bond, the stacking interaction between the π electrons of the furan aromatic rings can also determine the arrangement of the molecules in the dimers. The stacking interaction between aromatic rings can be realized as a sandwich, parallel displaced, edge-to-face or T-shaped arrangement of the aromatic molecules [36–39].

Two theoretical methods have been used to answer the question concerning the intermolecular interaction type. The first is the QTAIM [30], the second is the NCI method [32]. In the QTAIM method the presence of the bond path linking the interacting atoms [30] with the bond critical point (BCP) (ρ(r)) is evidence of an interaction. Higher electron density at the BCP indicates a stronger interaction. In the case of a very weak interaction, except the ρ(r) at the BCP, the nonlinearity of the bond path, ellipticity of the electron density at BCP (ε) and the distance of the BCP to the closest ring critical point (RCP) should also be taken into account. The bond path cannot be too curved, ellipticity cannot be too high and the BCP located at the bond path cannot be too close to the RCP. For hydrogen bonds the criteria for the existence of the weak CHO hydrogen bond were formulated [40, 41] but analogous criteria for other weak interactions are unknown.

The QTAIM theory also describes the energetic properties of the electrons at the BCP. Potential energy density of the electrons at the BCP (V(r)) expresses the pressure exerted on the electrons at the BCP by other electrons. Kinetic energy density (G(r)) is associated with the mobility of the electron density at the BCP and reflects the pressure exerted by the electron density cumulated at the BCP on other electrons [42, 43]. All these parameters for the investigated dimers are collected in Table 2 and the QTAIM graphs are presented in Figs. S5, S6, S7 and S8.

**Table 2:** Characteristics of the bond critical points (BCP) for the interactions in the dimers of furan, 2,3-dihydrofuran, 2,5-dihydrofuran and tetrahydrofuran

Relative energy^a^	Atoms	ρ(r) [a.u.]	ε(r)	d^b^ [Å]	V(r) [a.u.]	G(r) [a.u.]	H⋯O [Å]	CHO [°]
Furan								
0.00	H⋯O	0.0088	0.0783	0.0181	− 0.0053	0.0064	2.4484	140.560
	H⋯O	0.0088	0.0783	0.0181	− 0.0053	0.0064	2.4484	140.560
0.10	H⋯O	0.0062	0.2138	0.1234	− 0.0038	0.0047	2.7189	115.617
	H⋯C	0.0053	1.5300	0.2159	− 0.0028	0.0036		
	H⋯C	0.0053	1.5255	0.2159	− 0.0028	0.0036		
0.48	H⋯C	0.0065	1.9676	0.7604	− 0.0033	0.0042		
0.61	C⋯C	0.0060	3.7052	0.1840	− 0.0028	0.0035		
0.71	C⋯C	0.0064	0.0594	0.0351	− 0.0031	0.0040		
2,3-Dihydrofuran								
0.00	H⋯O	0.0087	0.0971	0.0244	− 0.0053	0.0060	2.5505	135.342
	H⋯O	0.0087	0.0972	0.0245	− 0.0053	0.0060	2.5505	135.342
	H⋯C	0.0061	0.6296	0.7513	− 0.0028	0.0034		
	H⋯C	0.0061	0.6304	0.7373	− 0.0028	0.0034		
0.03	H⋯O	0.0094	0.0816	0.0207	− 0.0057	0.0065	2.5013	136.933
	H⋯O	0.0094	0.0815	0.0207	− 0.0057	0.0065	2.5013	136.933
	H⋯C	0.0059	0.5103	0.6178	− 0.0026	0.0031		
	H⋯C	0.0059	0.5103	0.6176	− 0.0026	0.0031		
0.80	H⋯O	0.0110	0.0388	0.0132	− 0.0066	0.0076	2.3986	142.422
	H⋯O	0.0074	0.2942	0.0835	− 0.0048	0.0057	2.6442	122.143
	H⋯O	0.0076	0.1350	0.0491	− 0.0047	0.0054	2.6043	117.966
1.19	H⋯O	0.0102	0.0931	0.0163	− 0.0061	0.0075	1.6400	126.272
	H⋯O	0.0102	0.0930	0.0163	− 0.0061	0.0074	1.6400	126.272
1.30	H⋯O	0.0084	0.1307	0.0493	− 0.0052	0.0060	2.5681	127.797
	H⋯C	0.0063	0.3744	0.2722	− 0.0028	0.0035		
	H⋯C	0.0053	1.2489	0.4902	− 0.0027	0.0036		
	H⋯H	0.0048	1.8790	0.5759	− 0.0025	0.0033		
1.64	H⋯O	0.0098	0.0644	0.0289	− 0.0060	0.0068	2.4801	134.378
	H⋯C	0.0051	0.3464	0.1997	− 0.0023	0.0028		
	H⋯H	0.0048	0.7167	0.3480	− 0.0025	0.0033		
	H⋯H	0.0040	0.2887	0.3366	− 0.0022	0.0029		
2,5-Dihydrofuran								
0.00	H⋯O	0.0104	0.1097	0.0243	− 0.0065	0.0073	2.4692	132.429
	H⋯O	0.0107	0.1045	0.0215	− 0.0066	0.0074	2.4573	133.596
	H⋯C	0.0050	0.3301	0.2873	− 0.0022	0.0027		
	H⋯C	0.0050	0.3411	0.3339	− 0.0022	0.0026		
0.77	H⋯O	0.0117	0.0807	0.0233	− 0.0072	0.0082	2.3978	135.718
0.77	H⋯O	0.0122	0.0733	0.0156	− 0.0075	0.0085	2.3806	140.527
	H⋯C	0.0044	1.2436	0.3810	− 0.0020	0.0026		
	H⋯C	0.0044	1.2479	0.3814	− 0.0020	0.0026		
0.82	H⋯O	0.0111	0.1336	0.0423	− 0.0068	0.0078	2.4465	129.035
	H⋯C	0.0053	0.5346	0.6855	− 0.0023	0.0028		
	H⋯H	0.0035	0.7171	0.3975	− 0.0019	0.0025		
1.11	H⋯O	0.0099	0.0579	0.0218	− 0.0061	0.0071	2.4494	135.402
	H⋯O	0.0096	0.1058	0.0227	− 0.0059	0.0065	2.5264	131.376
	H⋯C	0.0039	0.2447	0.5118	− 0.0018	0.0022		
1.22	H⋯O	0.0060	0.7999	0.3047	− 0.0041	0.0049	2.9748	87.773
	H⋯O	0.0074	0.4633	0.4203	− 0.0049	0.0060	2.7557	99.558
Tetrahydrofuran								
0.00	H⋯O	0.0069	0.1052	0.0387	− 0.0045	0.0051	2.6988	125.131
	H⋯O	0.0100	0.1299	0.0250	− 0.0060	0.0069	2.4754	137.636
	H⋯O	0.0111	0.0444	0.0090	− 0.0068	0.0076	2.4293	143.184
	H⋯H	0.0037	2.0328	0.2626	− 0.0020	0.0026		
0.06	H⋯O	0.0111	0.0629	0.0041	− 0.0067	0.0074	2.4182	156.946
	H⋯O	0.0105	0.0710	0.0171	− 0.0063	0.0071	2.4562	148.966
	H⋯H	0.0045	0.1561	0.0715	− 0.0023	0.0028		
	H⋯H	0.0049	0.2065	0.1366	− 0.0026	0.0033		
	H⋯H	0.0037	2.1208	0.6785	− 0.0021	0.0027		
0.10	H⋯O	0.0112	0.0461	0.0135	− 0.0070	0.0078	2.4301	137.183
	H⋯O	0.0057	0.7396	0.2668	− 0.0039	0.0047	2.8688	105.805
	H⋯O	0.0083	0.0917	0.0668	− 0.0052	0.0059	2.5957	126.335
	H⋯O	0.0061	0.0696	0.0741	− 0.0040	0.0046	2.7173	122.740
	H⋯H	0.0040	0.3203	0.1197	− 0.0021	0.0026		
0.14	H⋯O	0.0109	0.0514	0.0085	− 0.0067	0.0074	2.4420	142.027
	H⋯O	0.0095	0.0808	0.0383	− 0.0058	0.0066	2.5383	132.523
	H⋯H	0.0043	0.3021	0.3243	− 0.0025	0.0032		
	H⋯H	0.0045	0.2951	0.0867	− 0.0023	0.0028		
0.33	H⋯O	0.0061	0.5540	0.2169	− 0.0037	0.0045	2.7202	109.953
	H⋯O	0.0068	0.3676	0.1949	− 0.0043	0.0051		
	H⋯H	0.0036	0.4409	0.2533	− 0.0019	0.0025		
	H⋯H	0.0037	0.3774	0.3142	− 0.0019	0.0025		
	H⋯H	0.0043	0.1855	0.2943	− 0.0024	0.0032		
	H⋯H	0.0045	0.0712	0.1125	− 0.0024	0.0030		
1.29	H⋯O	0.0109	0.0877	0.0146	− 0.0066	0.0074	2.4407	143.984
	H⋯H	0.0047	0.3370	0.1505	− 0.0024	0.0030		
	H⋯H	0.0043	0.1019	0.0619	− 0.0022	0.0027		
1.64	H⋯O	0.0074	0.2230	0.0441	− 0.0047	0.0053	2.6601	126.962
	H⋯O	0.0080	0.1908	0.0341	− 0.0048	0.0055		
	H⋯H	0.0039	0.4176	0.1311	− 0.0021	0.0026		
	H⋯H	0.0040	0.0611	0.0821	− 0.0021	0.0026		
	H⋯H	0.0046	0.4227	0.1918	− 0.0025	0.0031		
	H⋯H	0.0050	0.2170	0.0600	− 0.0025	0.0030		
1.89	H⋯O	0.0104	0.0587	0.0211	− 0.0064	0.0072	2.4407	143.984
	H⋯O	0.0070	0.2376	0.1366	− 0.0045	0.0053	2.6928	116.312
	H⋯O	0.0072	0.2460	0.1607	− 0.0047	0.0056	2.6828	113.742
	H⋯H	0.0051	0.2608	0.1237	− 0.0028	0.0034		
	H⋯H	0.0051	0.2210	0.0614	− 0.0027	0.0032		

^a^Relative energy refers to the structures in Figs. 1, 2, 3 and 4

^b^d—the difference between the length of the bond path and the distance of the atoms linked by the bond path

In Table 1 are listed all the intermolecular interactions for which the bond paths with the BCP have been found. The interactions that bind the molecules in the dimers are the C–H⋯O and H⋯H, and the main interaction linking the molecules in furan dimers seems to be the C–H⋯O hydrogen bond. The C⋯C and H⋯C bond paths are typical for the stacking of the aromatic molecules [44]. It could be expected that many CH bonds linked to the aromatic and aliphatic ring represent the C–H⋯π interaction that determines a mutual orientation of the molecules in dimer [45].

The CH⋯O hydrogen bond is significantly weaker than the strongest OHO or OHN hydrogen bonds—the classical hydrogen bond investigated using many experimental methods [46–48]. The CH⋯O hydrogen bond is experimentally less evident and it took a longer time to exhibit the existence and importance of this type of interaction [49]. The low strength of the CH⋯O hydrogen bond is the reason why this interaction was characterized as an improper hydrogen bond [50–52] with the strength comparable to the stacking and other very weak intermolecular interactions.

Although the electron density at BCP is an evidence of interaction, the presence of the bond path with the BCP is not a sufficient criterion for the existence of the hydrogen bond and other criteria of hydrogen bond existence must be taken into account. Interactions in furan dimers must be analysed including other criteria that are particularly important in the case of very low electron density at the BCP. Table 1 includes the QTAIM parameters which are necessary to confirm the existence of a weak intermolecular interaction. High ellipticity of the electron density at the BCP and nonlinearity of the bond path is the reason why the majority of the interactions in Table 2 do not meet the criteria for the hydrogen bond. Except the high ellipticity and nonlinearity of the bonds, instability of the listed interactions is also expressed by the balance between potential and kinetic energy density at the BCP. For a few dimers, for example these represented by the energies of 0.10, 0.48 and 0.61 for furan, the elimination of the interactions which do not fulfil the criteria for a hydrogen bond leads to dimers being formed without any interaction binding the furan molecules together.

Classification of the C–H⋯O interactions in furan, for example as the CHO hydrogen bond or a weak van der Waals interaction, is associated with a general definition of the hydrogen bond. In accordance with a very broad hydrogen bond definition [53, 54], all the interactions can be treated as a hydrogen bond. Using the hydrogen bond definition developed within the frame of the QTAIM approach [30], only the strongest CHO in the cyclic furan dimer can be found as the CHO hydrogen bond.

The second popular interaction in the furan dimers is the H⋯H which seems to be a dihydrogen bond. When comparing the electron density at the BCP with the electron density of the classical dihydrogen bond [55], it appears that the electron density for H⋯H interactions in Table 2 is one order of magnitude fewer. Additionally, the H⋯H interaction in the investigated furans is characterized by high ellipticity. Because the H⋯H contact with a short H⋯H distance is not necessarily a dihydrogen bond but may also be a van der Waals interaction [56–58] taking into account the QTAIM parameters in Table 2 it is evident that the H⋯H contacts belong rather to very weak contacts and do not exhibit the features of a dihydrogen bond.

The second theoretical method used in this study to characterize the interaction in the furan dimers is the NCI approach [32]. It is very useful for investigating very weak interactions such as van der Waals, hydrogen bonds, steric repulsion and dispersion. The main parameter used in the frame of the NCI method, is reduced electron density gradient of the electron density which describes the deviation from a homologous electron density distribution *s* = 1/(2(3*π*^2^)^1/3^)|∇ρ|/ρ^4/3^. The reduced gradient of electron density is very small, close to zero for a covalent bonding but is very high with positive values in the regions far from the molecule where the electron density exponentially decays to zero. The efficiency of the NCI method is a visualization of the interaction in the plots of the reduced density gradient versus the electron density multiplied by the sign of the second Hessian eigenvalue (λ_2_) of electron density that makes it possible to differentiate a repulsive and attractive interaction. The location of the spikes in the interaction plot—a hydrogen bond at higher density values (− 0.01 < ρ < − 0.05 a.u.) and dispersion interactions at lower density values (ρ < − 0.01 a.u.)—differentiates repulsive and attractive interactions. Another visualization method is to draw the gradient isosurfaces in the real space for the molecule in colours traditionally used in the NCI approach: blue for attractive, red for repulsive and green for intermediate strength interactions.

**Fig. 5:**
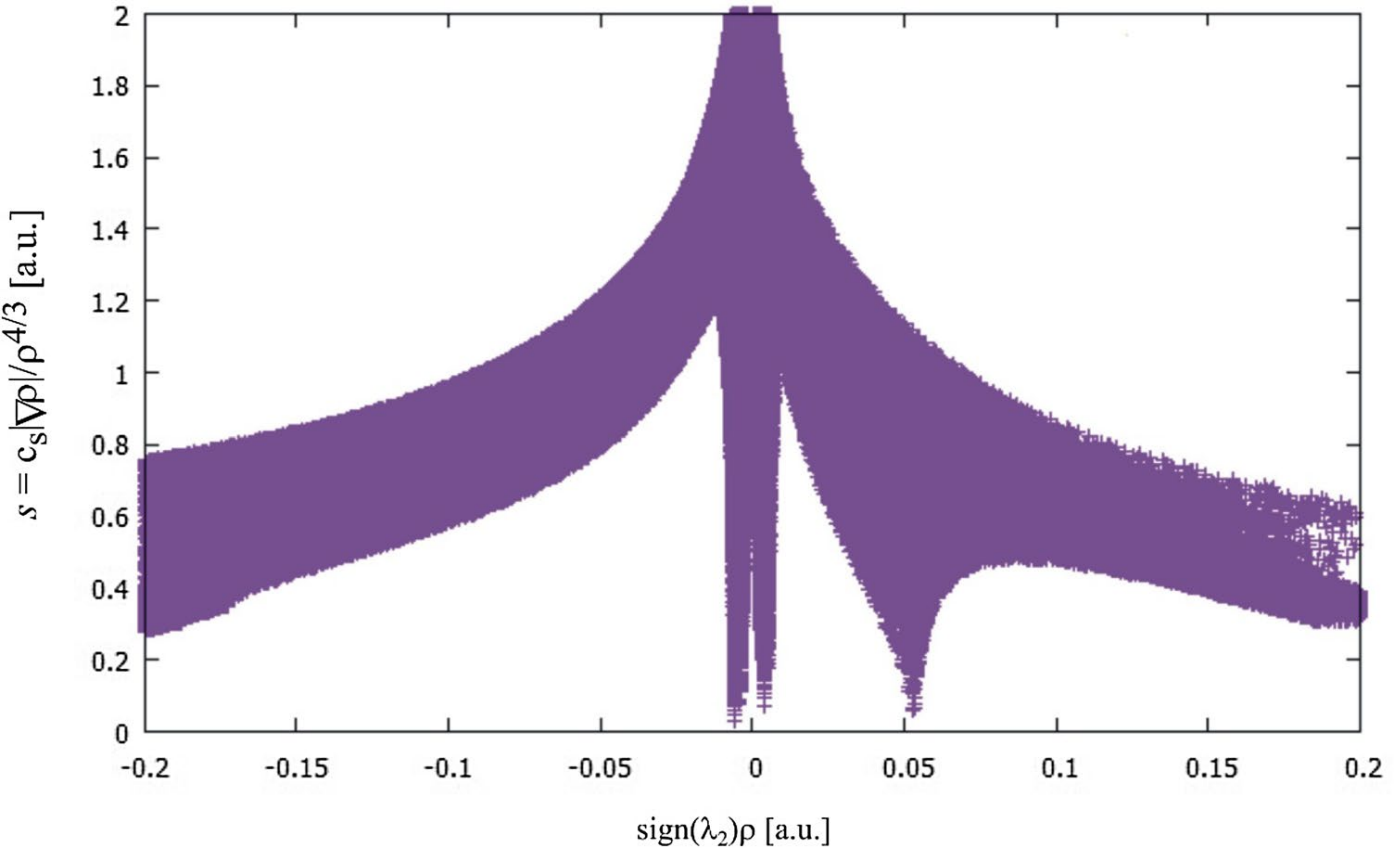
NCI plots of the reduced density gradient versus the electron density multiplied by the sign of λ_2_ for the investigated dimers. The spikes at about 0 refer to dispersive interactions, the spike at about 0.05 a.u. illustrates the repulsive interaction in the center of the monomers

The plots of the reduced density gradient versus the electron density multiplied by the sign of the second Hessian eigenvalue for all the investigated furan dimers are very similar to each other and may be represented by the plot depicted in Fig. 5. The gradient isosurfaces in the real space for the investigated dimers are shown in Figs. S9, S10, S11 and S12.

The NCI approach answers the question concerning the type of interactions linking the monomers in the investigated dimers. All of them, even the C–H⋯O hydrogen bonds in the cyclic dimers, have character of a weak, dispersive interaction and suggests that the main interaction between the furan molecules in many of them is the stacking of the rings.

### Decomposition of the bonding energy

To interpret the bonding force linking furans in the investigated dimers, the energy decomposition according to Morokuma–Ziegler has been performed [34, 35]. In this approach the bonding energy is decomposed into electrostatic (E_elect_), Pauli (E_Pauli_), orbital (E_orb_) and dispersive (E_dysp_) component.

$${{\text{E}}_{{\text{bonding}}}}={{\text{E}}_{{\text{elect}}}}+{{\text{E}}_{{\text{Pauli}}}}+{{\text{E}}_{{\text{orb}}}}+{{\text{E}}_{{\text{dysp}}}}$$

E_elect_ is the direct Coulomb interaction between the unperturbed charge of the two monomers. E_Pauli_ expresses the destabilizing Pauli repulsion between the occupied orbitals. E_orb_ illustrates the interaction energy between the orbitals of the complex components. E_dysp_ is the dispersion energy of the intermolecular van der Waals interaction. The sum of E_elect_ and E_Pauli_ represents the steric interaction.

The total interaction energy is the balance between the steric repulsion, dispersion and orbital interaction. The characteristic feature is that among the energy components in Table 3 the dispersive energy has a significant contribution similar to the electrostatic interaction even for the dimers of aliphatic tetrahydrofuran. Either the orbital interaction cannot be neglected as an additional binding force linking two monomers of the furan derivatives.

**Table 3:** Decompositions of the interaction energy of the investigated furan dimers expressed in kcal/mol

Relative energy^a^	E_elect_	E_Pauli_	E_steric_	E_orb_	E_dysp_	E_total_
Furan						
0.00	− 3.00	3.09	0.09	− 1.13	− 2.07	− 3.11
0.10	− 2.12	3.15	1.03	− 1.08	− 3.13	− 3.18
0.48	− 1.80	2.91	1.11	− 1.16	− 2.78	− 2.82
0.61	− 2.31	4.43	2.12	− 1.04	− 3.66	− 2.58
0.71	− 2.25	4.43	2.18	− 0.99	− 3.71	− 2.52
2,3-Dihydrofuran						
0.00	− 4.40	6.13	1.73	− 1.99	− 5.25	− 5.51
0.03	− 4.70	6.27	1.57	− 1.96	− 5.17	− 5.56
0.80	− 4.10	4.81	0.71	− 1.58	− 3.55	− 4.41
1.19	− 45.06	111.9	66.84	− 24.52	− 3.35	38.97
1.30	− 3.52	5.01	1.49	− 1.44	− 3.9	− 3.86
1.64	− 2.53	4.25	1.72	− 1.29	− 4.09	− 3.66
2,5-Dihydrofuran						
0.00	− 4.62	6.40	1.78	− 1.87	− 4.98	− 5.07
0.77	− 3.70	5.06	1.36	− 1.55	− 3.94	− 4.13
0.77	− 3.32	4.88	1.56	− 1.52	− 4.19	− 4.16
0.82	− 4.56	4.50	− 0.06	− 2.69	− 4.04	− 6.79
1.11	− 3.49	4.90	1.41	− 1.57	− 3.77	− 3.93
1.22	− 2.63	4.41	1.78	− 1.38	− 4.20	− 3.80
Tetrahydrofuran						
0.00	− 4.78	6.43	1.65	− 2.04	− 4.75	− 5.14
0.06	− 4.45	6.46	2.01	− 2.17	− 4.75	− 5.37
0.10	− 41.04	95.37	54.33	− 23.33	− 3.68	27.32
0.14	− 4.28	6.14	1.86	− 2.03	− 4.99	− 5.17
0.33	− 2.12	4.24	2.12	− 1.24	− 4.36	− 3.48
1.29	− 2.74	4.31	1.57	− 1.42	− 3.84	− 3.70
1.64	− 2.52	4.91	2.39	− 1.59	− 4.56	− 3.77
1.84	− 4.05	6.01	1.96	− 1.93	− 5.12	− 5.10

^a^Relative energy refers to the structures in Figs. 1, 2, 3 and 4

Decomposition energy exhibits instability of the cyclic dimers of 2,3-dihydrofuran and the structure of tetrahydrofuran with the energy of 0.10 kcal/mol which is a bit similar to the cyclic structure. Despite the stability of these structures confirmed by positive vibrational frequencies, the steric interaction, expressed as the sum of electrostatic and Pauli interaction, is very great and the sign of the total energy expresses instability of these complexes. Stability of the furan dimers is a balance between stabilizing effect of dispersive and orbital interaction and the steric effect in which repulsive interaction between the furan molecules dominates. The steric interaction in furan dimers is represented by the intensive spike at about 0.05 a.u. in the plot of the reduced density gradient versus the electron density multiplied by the sign of the second Hessian eigenvalue (Fig. 5). The spikes close to zero, especially not very intense for 2,3-dihydrofuran, are related to dispersion and are responsible for stabilization of the dimers. Also comparison of the electron density gradient isosurfaces in real space for 2,3-dihydrofuran dimers (Fig. S10) illustrates that for the cyclic structure the dispersive interaction almost disappears. For the unstable dimers the total energy does not correlate with the complexation energy obtained in the optimization with Gaussian. For other furan dimers both energies are linked by a linear correlation (complexation energy = 1.0522E_total_ + 0.4449, R^2^ = 0.9946). Due to the steric interaction, the dimer of 2,3-dihydrofuran with a relative energy of 1.19 kcal/mol and the dimer of tetrahydrofuran with a relative energy of 0.10 kcal/mol cannot be stable because of the steric interaction even if the positive vibrational frequencies confirm the stability of these structures. The cyclic structure with two CHO hydrogen bonds can be realized for furan but is unstable for 2,3-dihydrofuran and is not possible for 2,5-dihydrofuran and tetrahydrofuran.

The general conclusion drawn from the energy decomposition is importance of the dispersive interaction comparable to the electrostatic attraction. According to Figs. S1, S2, S3 and S4, the electrostatic potential seems to be a sufficient explanation of the binding forces but the analysis of the energy components exhibits the role of the dispersion and interorbital interaction. Importance of dispersive interaction as a binding force linking the molecules together was also evidenced for other intermolecular complexes, for example the complex of furan–indol [59]. The investigated furan dimers confirm the role of dispersive interaction which can compete with very weak hydrogen bonds.

## Conclusions

1. The dimers of furan 2,3-dihydrofuran, 2,5-dihydrofuran and tetrahydrofuran can be formed by weak interactions. Even if the cyclic dimer formed by two CHO hydrogen bonds may be present, its strength is comparable with the stacking. Among the furan dimers there is not any with the single CH⋯O and CH⋯π hydrogen bond. Typical dimers of furan, 2,3-dihydrofuran, 2,5-dihydrofuran and tetrahydrofuran are determined by the stacking interaction.
2. The electrostatic potential and the energy decomposition performed for the low energy dimers suggests that the force linking the investigated molecules into dimers is the electrostatic and dispersive interaction which for some dimers is comparable.
3. For some dimers the interactions linking the furan molecules together are so weak, that detailed QTAIM analysis does not find any interaction responsible for dimer formation. Very sensitive NCI method exhibit the role of dispersive interactions which are crucial for the formation of furan dimers.

## Electronic supplementary material

Below is the link to the electronic supplementary material.

Supplementary material 1 (DOCX 2718 KB)
