# Supplementary material for: Weak interactions in furan dimers
Source: J Comput Aided Mol Des. 2018 Sep 14;32(11):1247–58. doi: 10.1007/s10822-018-0163-5 (PMC6267657; doi:10.1007/s10822-018-0163-5)
Supplement: Supplementary file 1 — Supplementary material 1 (DOCX 2718 KB) [file 10822_2018_163_MOESM1_ESM.docx]

**Supporting information**


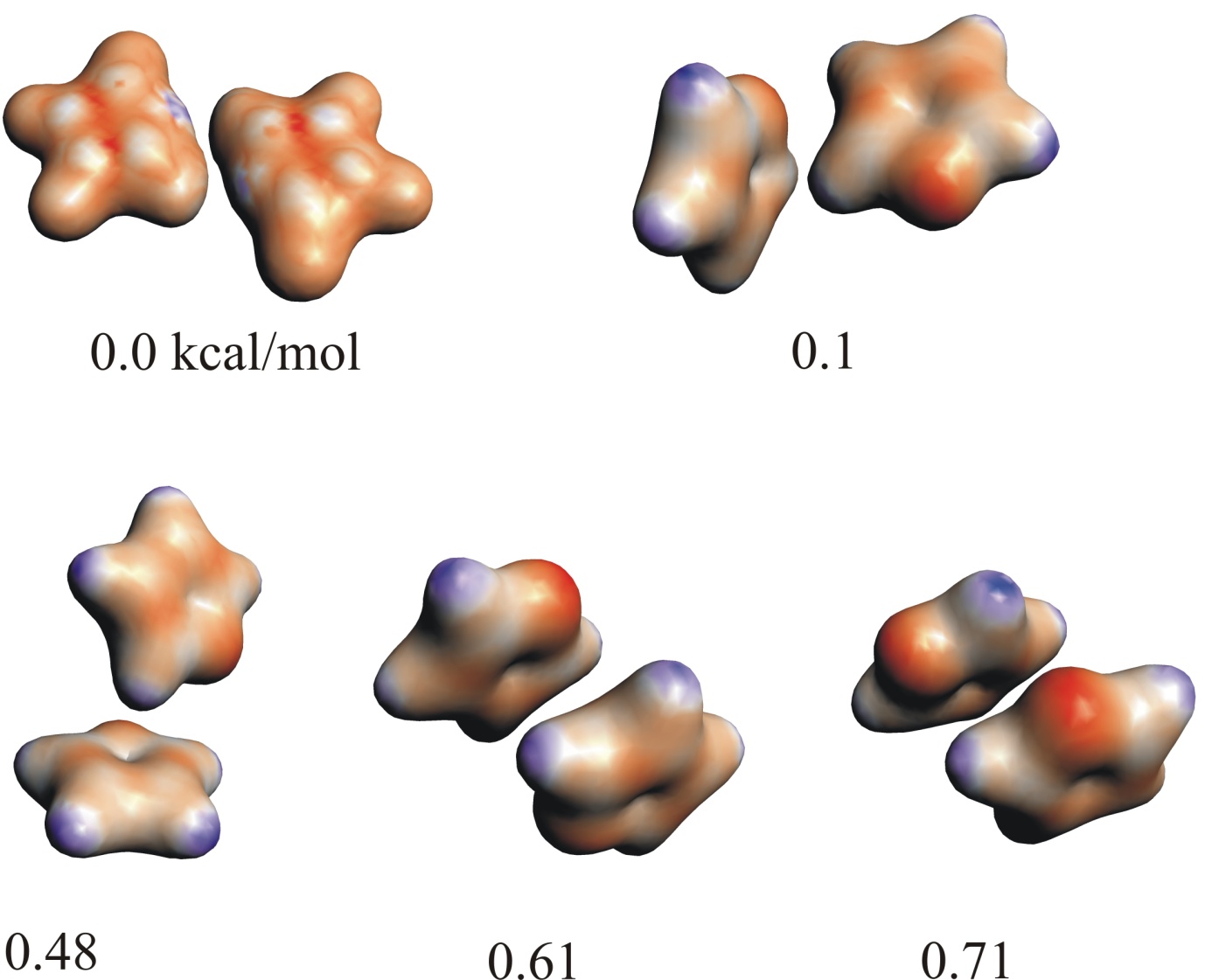


Fig. S1. Electrostatic potential for furan dimers characterized by relative energy in kcal/mol. Purple – positive, orange – negative.


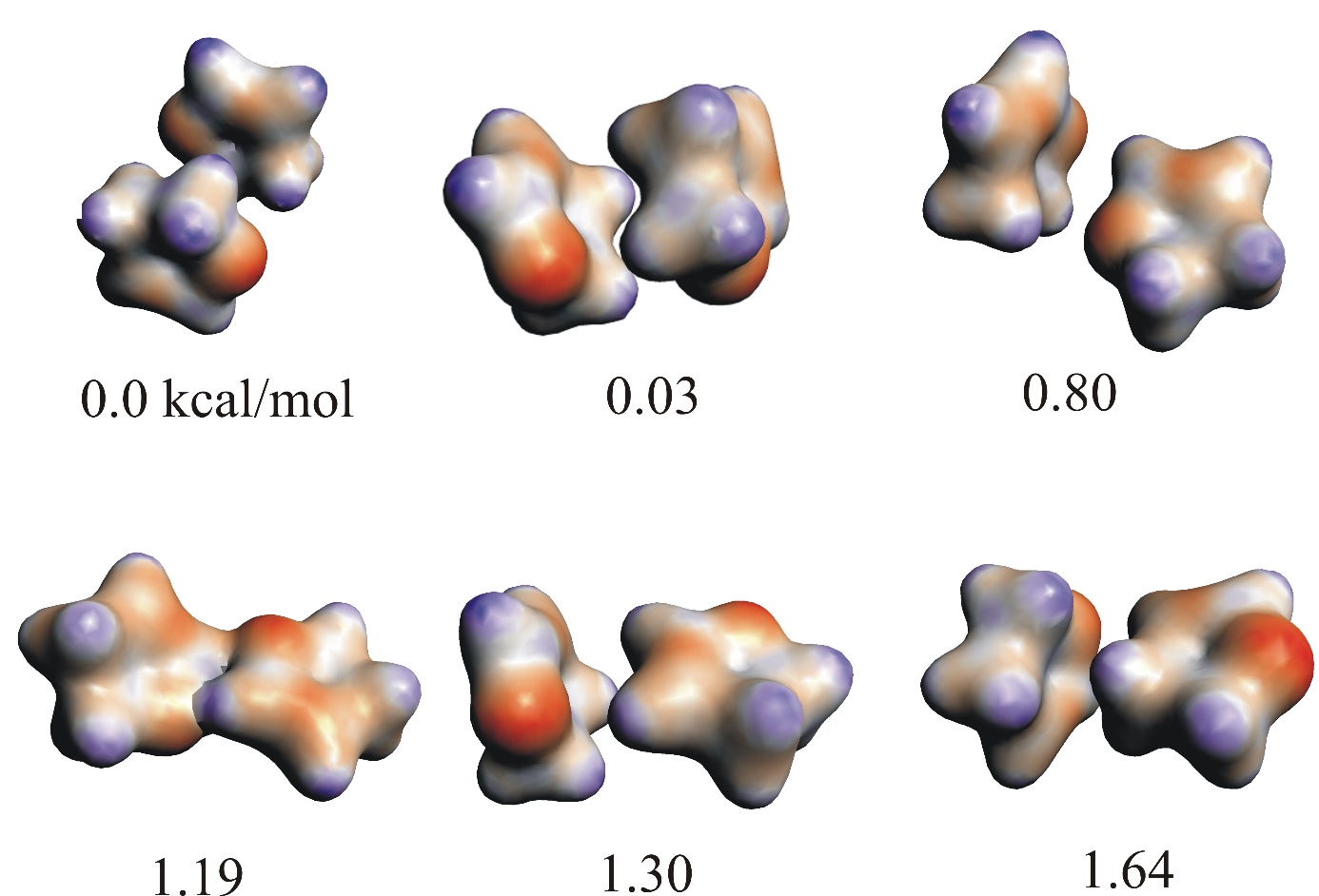


Fig. S2. Electrostatic potential for 2,3-dihydrofuran dimers characterized by relative energy in kcal/mol. Purple – positive, orange – negative.


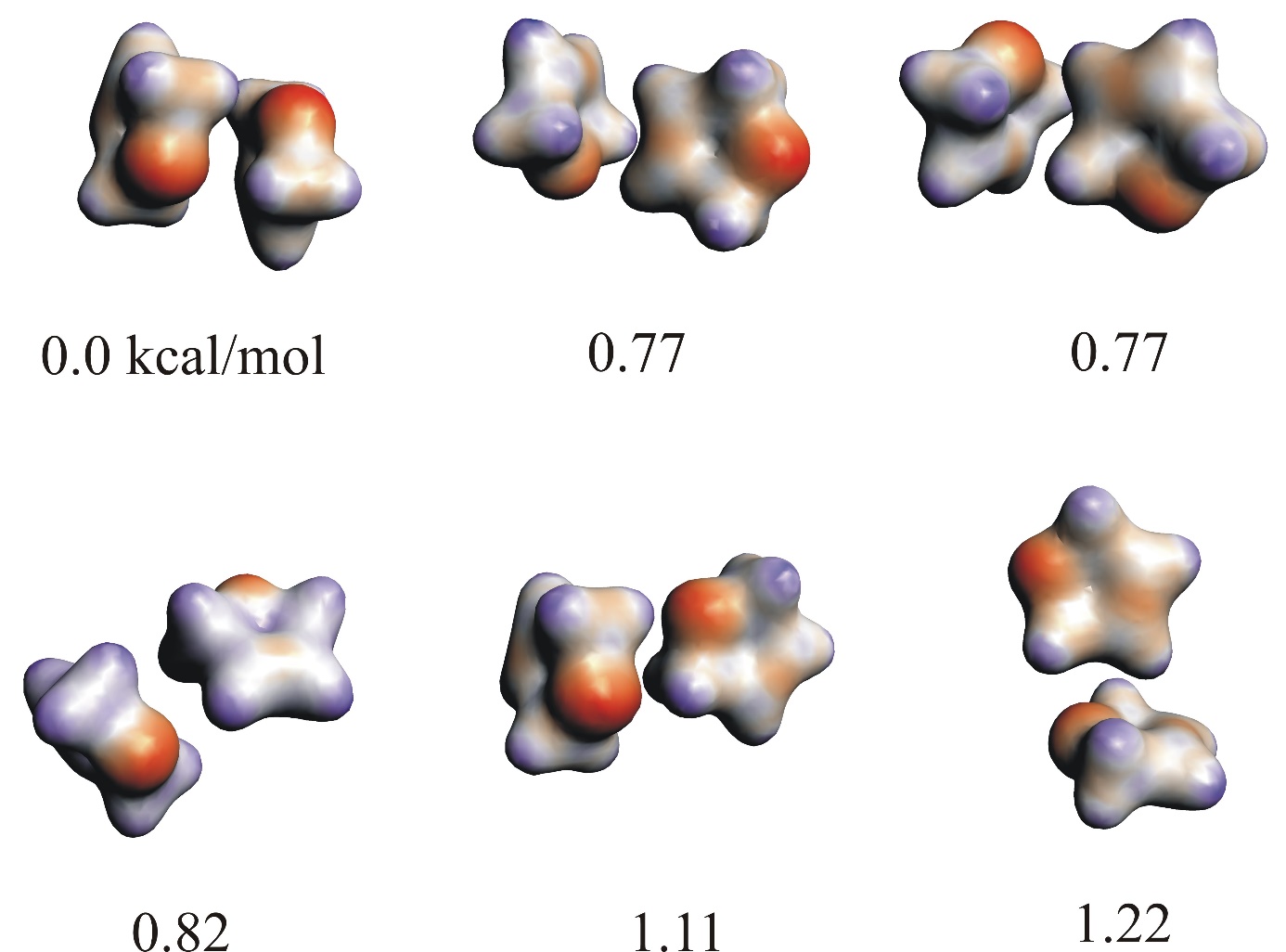


Fig. S3. Electrostatic potential for 2,5-dihydrofuran dimers characterized by relative energy in kcal/mol. Purple – positive, orange – negative.


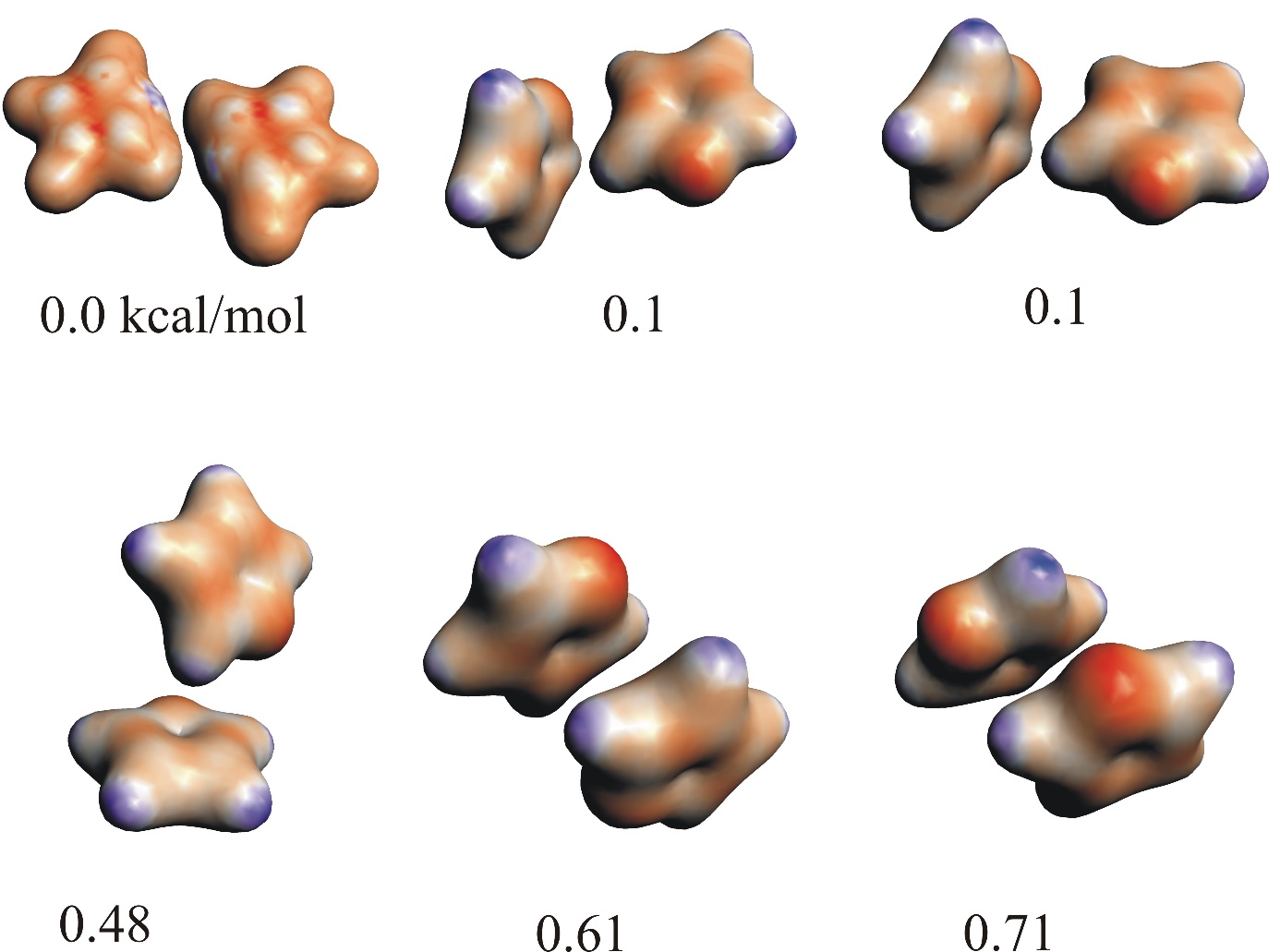


Fig. S3. Electrostatic potential for 2,5-dihydrofuran dimers characterized by relative energy in kcal/mol. Purple – positive, orange – negative.


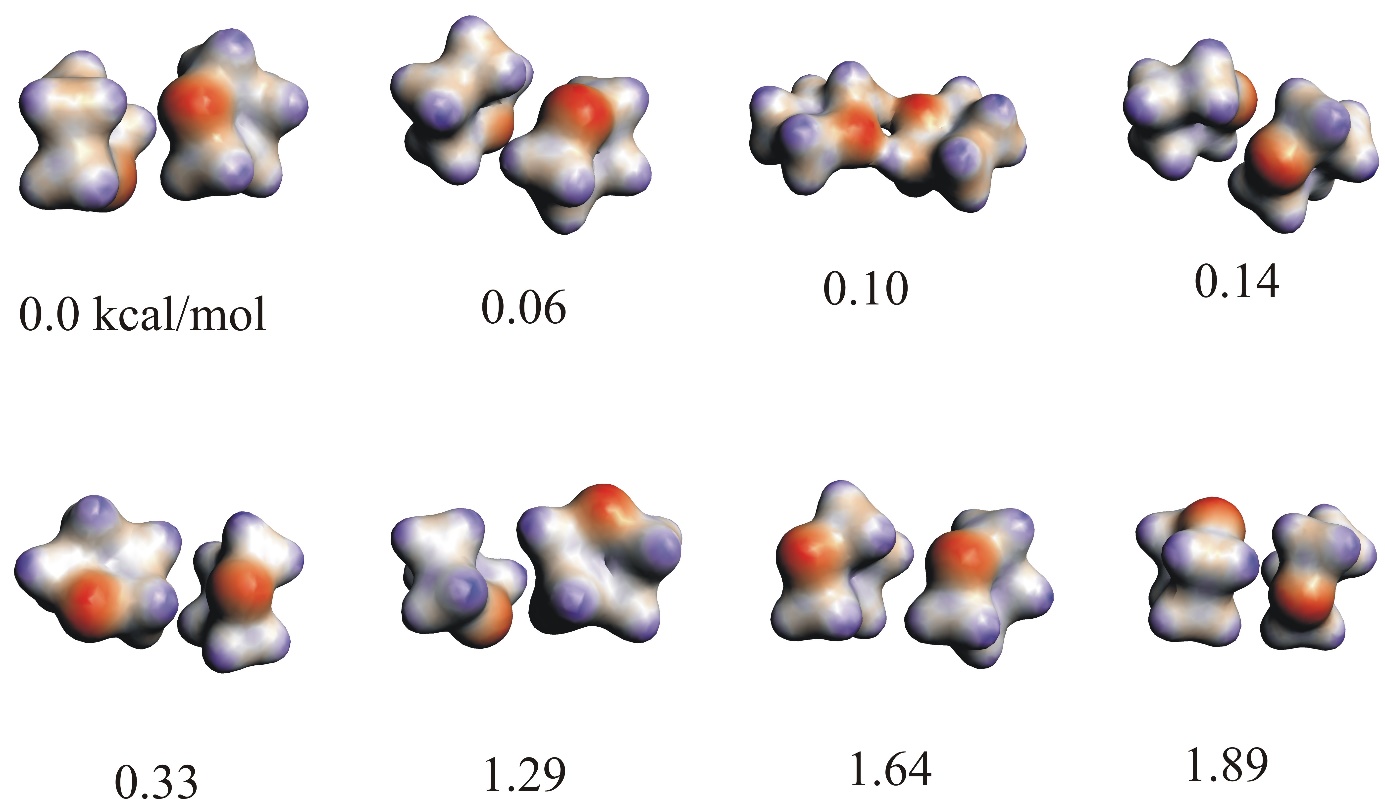


Fig. S4. Electrostatic potential for tetrahydrofuran dimers characterized by relative energy in kcal/mol. Purple – positive, orange – negative.


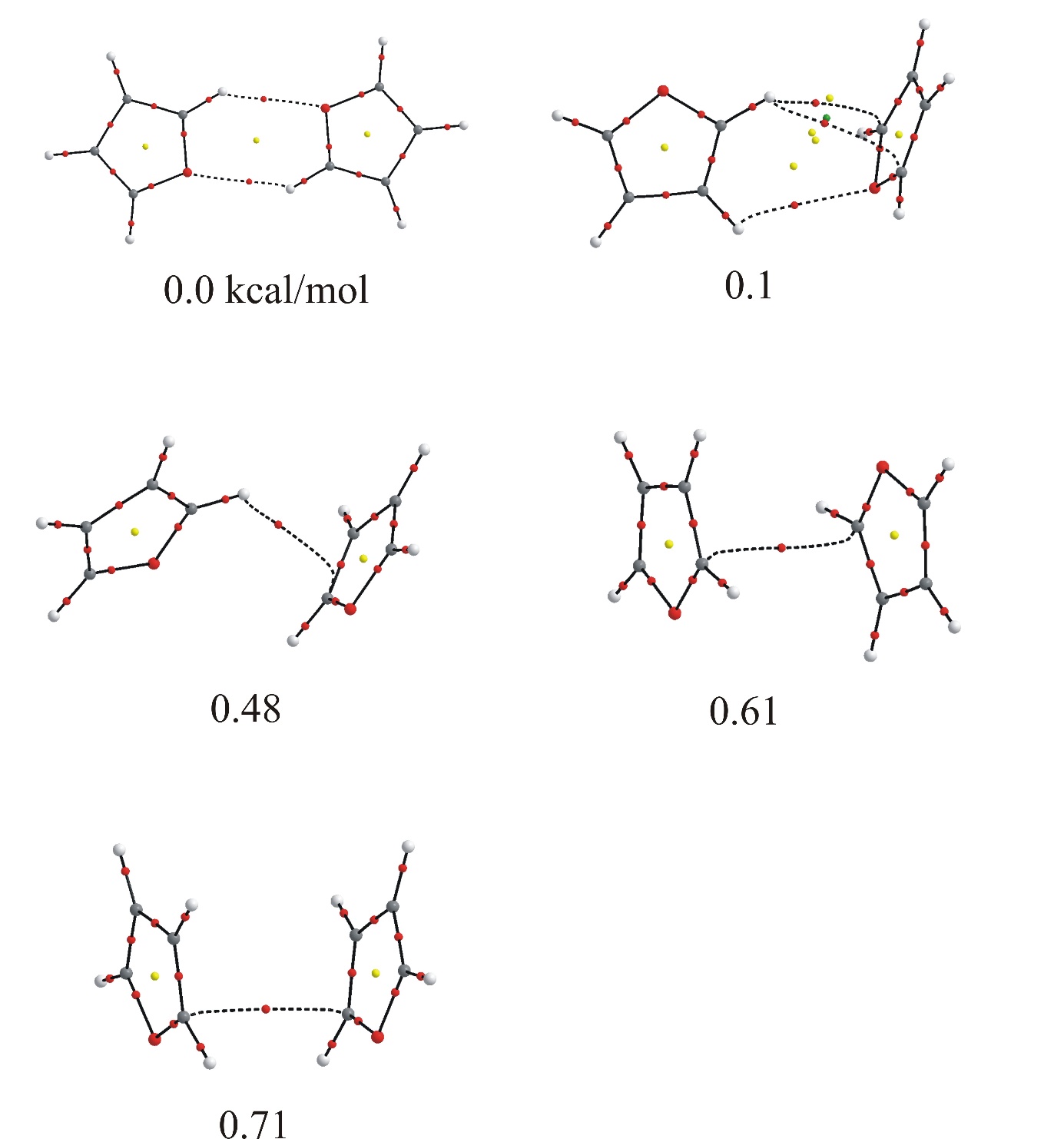


Fig. S5. Molecular graphs of furan dimers characterized by relative energy in kcal/mol. Carbon, hydrogen, nitrogen, oxygen and sodium atoms are marked as grey, white, blue, red and purple dots respectively. Red yellow and green dots represent bond critical points - BCPs, ring critical points - RCPs and cage critical points - CCP respectively.


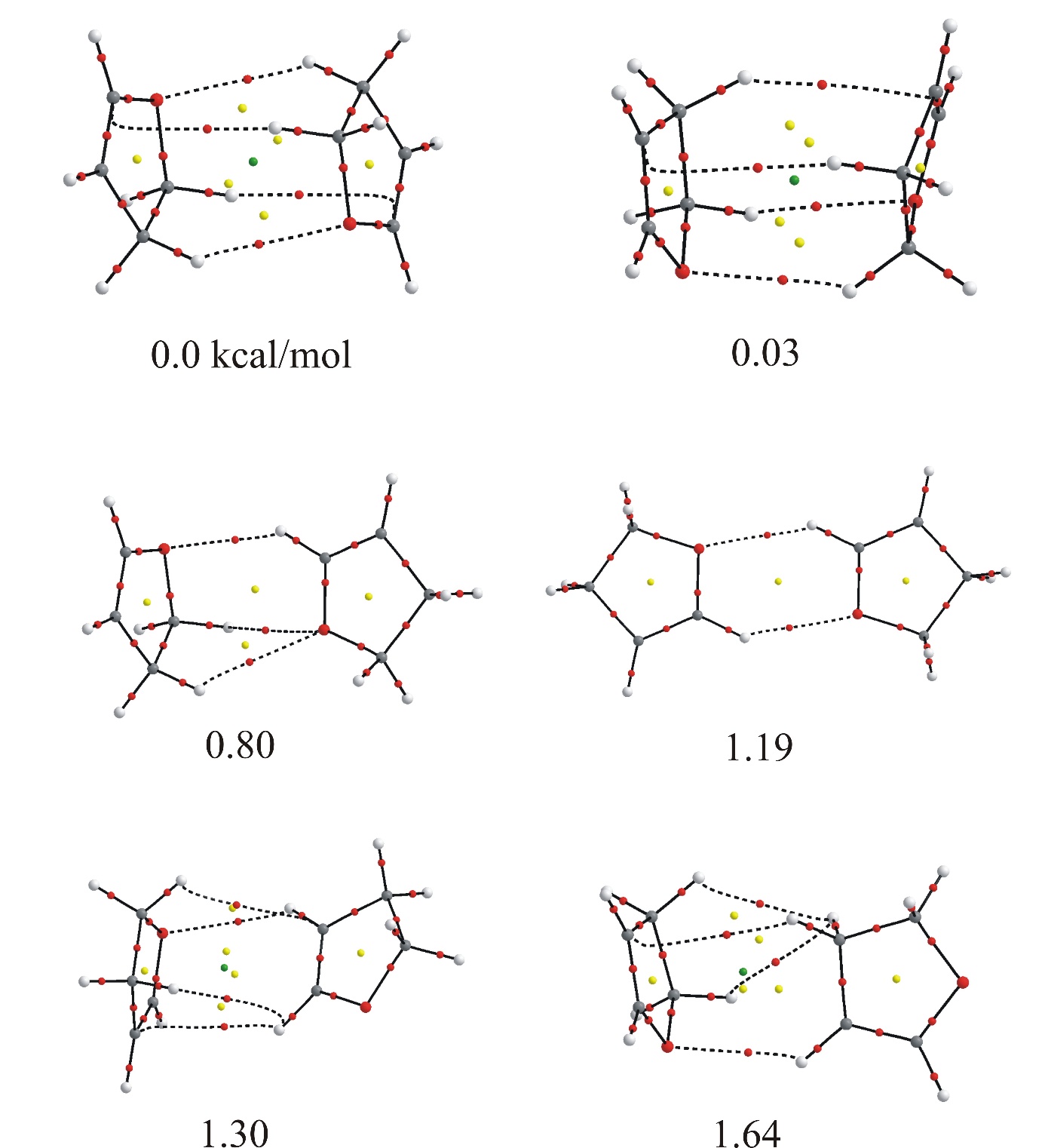


Fig. S6. Molecular graphs of 2,3-dihydrofuran dimers characterized by relative energy in kcal/mol. Carbon, hydrogen, nitrogen, oxygen and sodium atoms are marked as grey, white, blue, red and purple dots respectively. Red yellow and green dots represent bond critical points - BCPs, ring critical points - RCPs and cage critical points - CCP respectively.


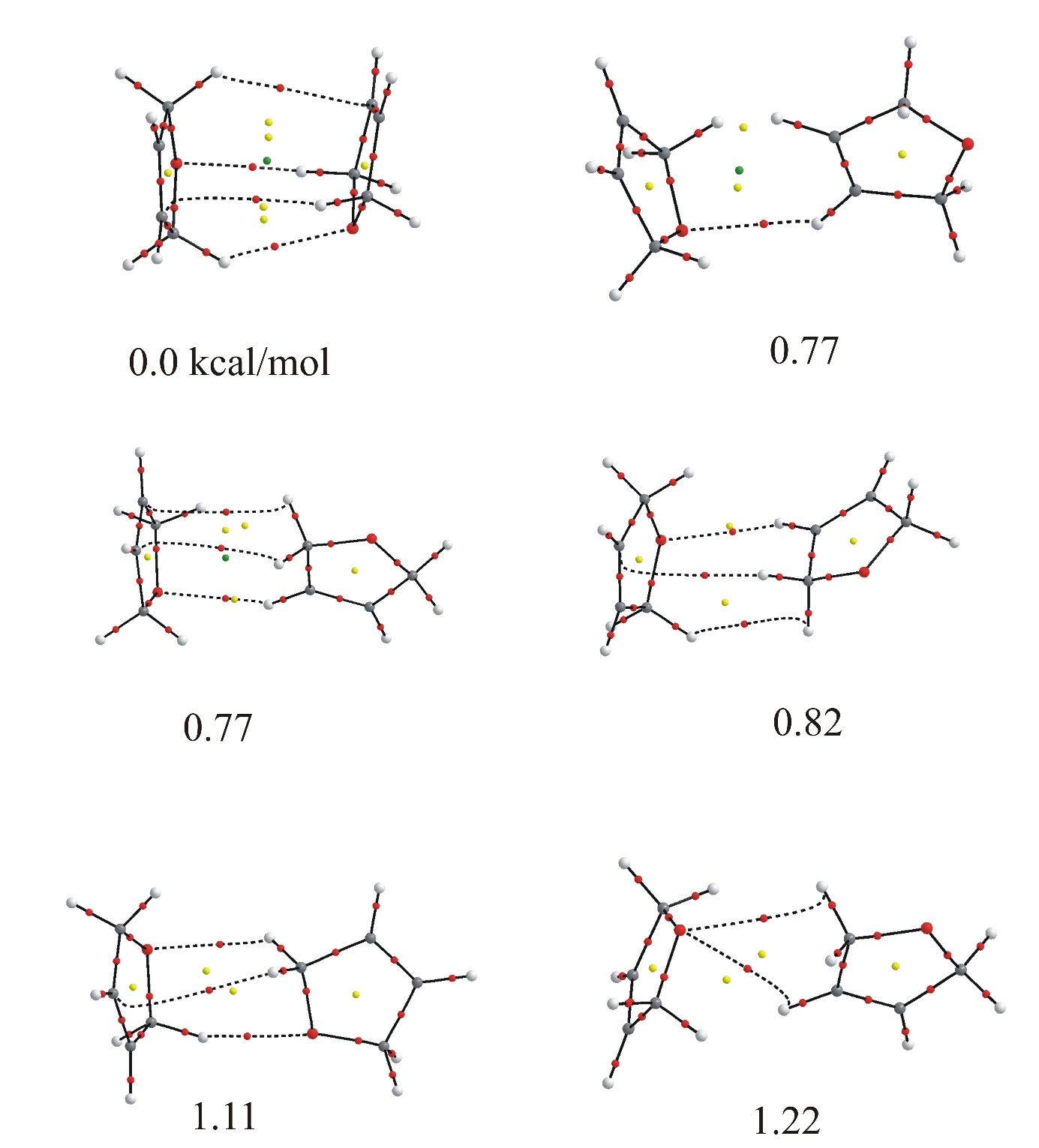


Fig. S7. Molecular graphs of 2,5-dihydrofuran dimers characterized by relative energy in kcal/mol. Carbon, hydrogen, nitrogen, oxygen and sodium atoms are marked as grey, white, blue, red and purple dots respectively. Red yellow and green dots represent bond critical points - BCPs, ring critical points - RCPs and cage critical points - CCP respectively.


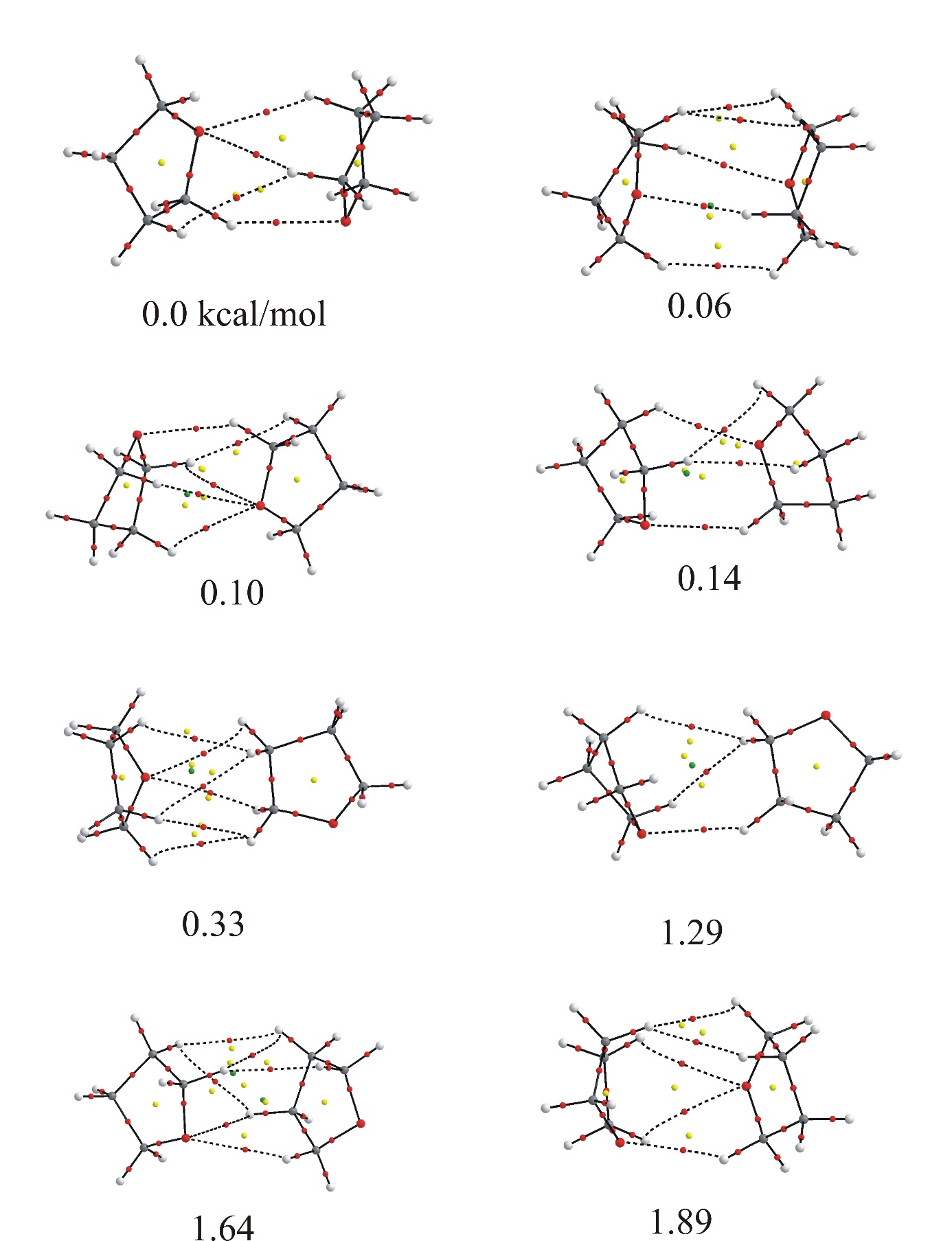


Fig. S8. Molecular graphs of tetrahydrofuran dimers characterized by relative energy in kcal/mol. Carbon, hydrogen, nitrogen, oxygen and sodium atoms are marked as grey, white, blue, red and purple dots respectively. Red yellow and green dots represent bond critical points - BCPs, ring critical points - RCPs and cage critical points - CCP respectively.


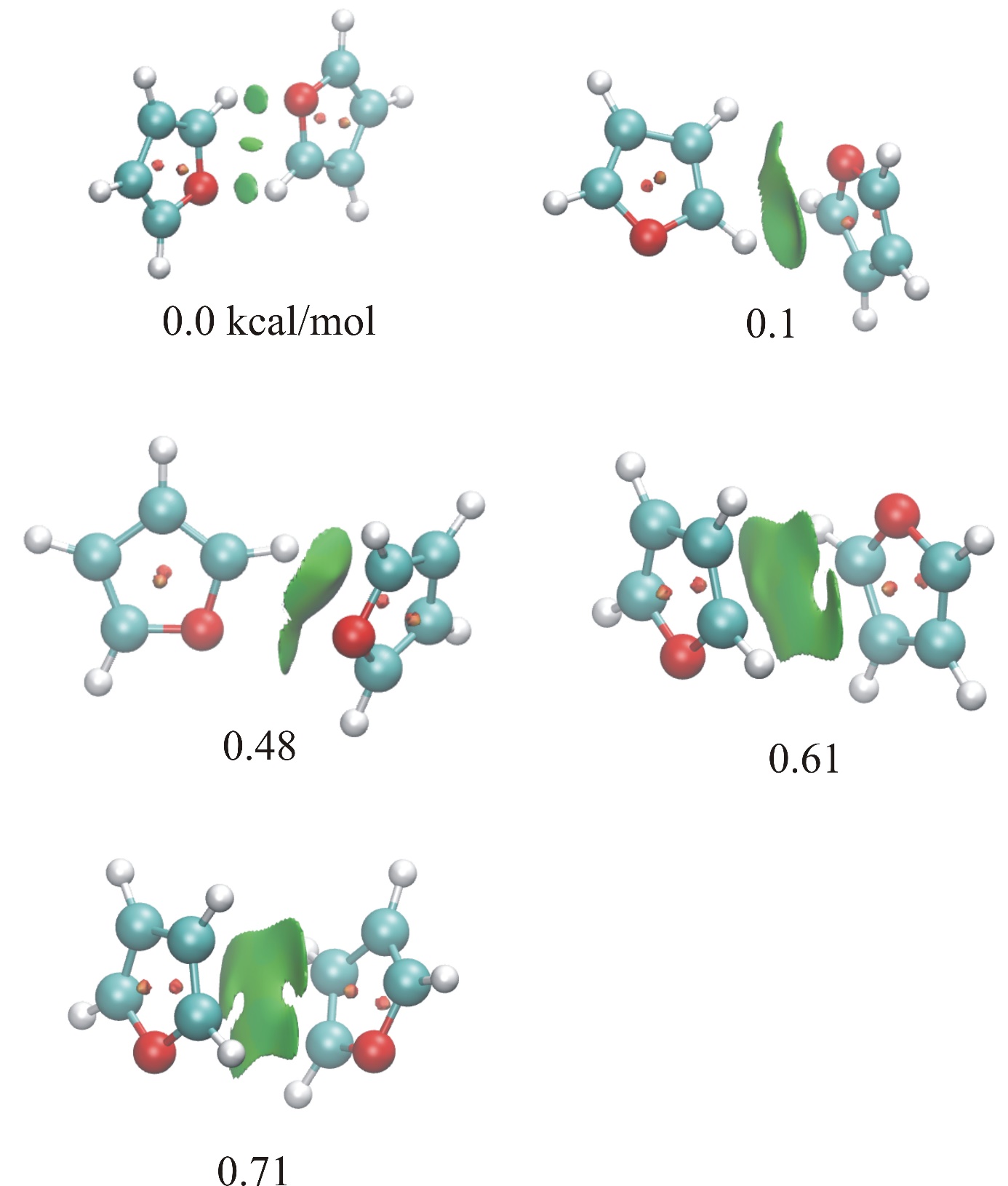


Fig. S9. NCI surfaces for furan dimers.


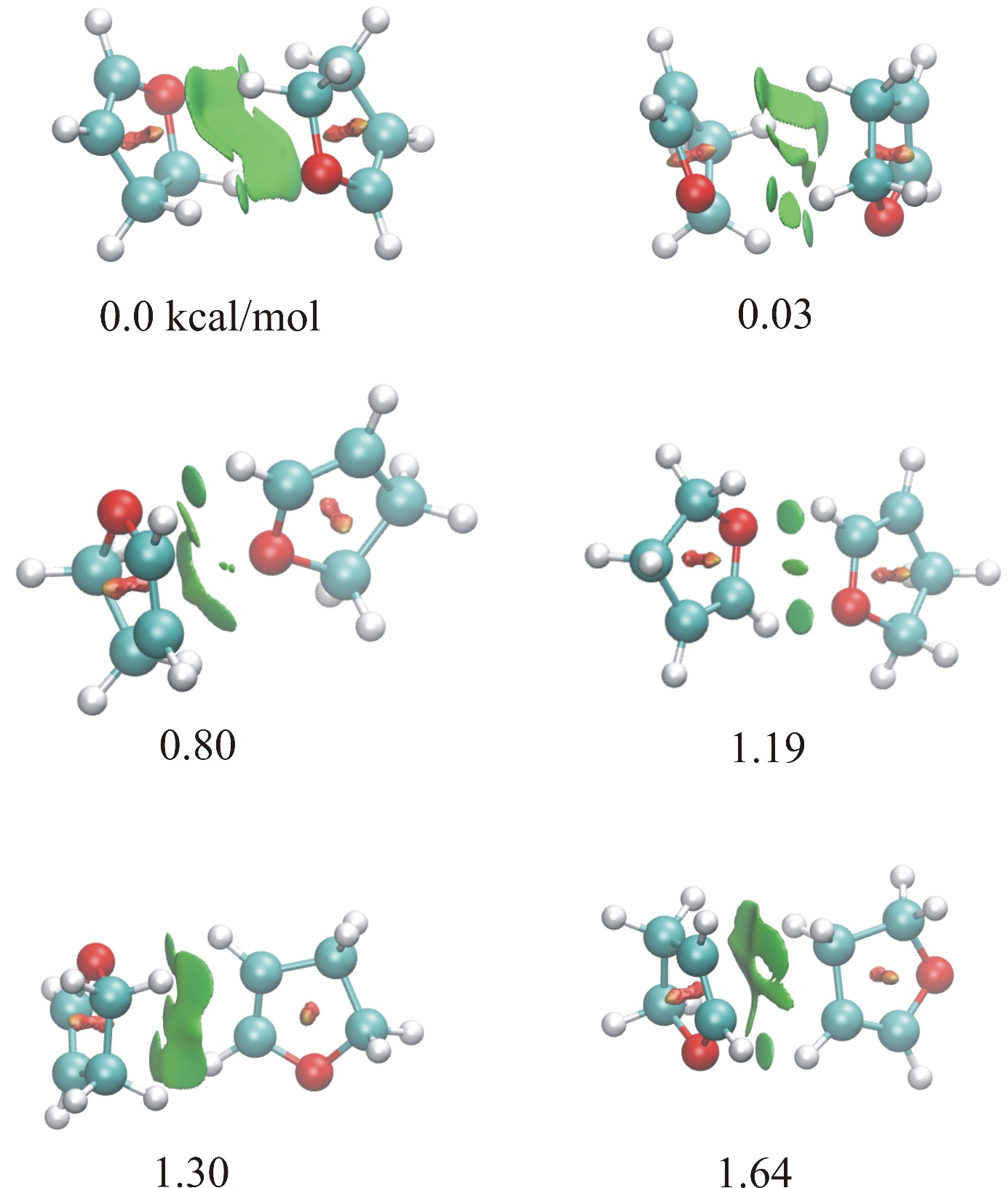


Fig. S10. NCI surfaces for 2,3-dihydrofuran dimers.


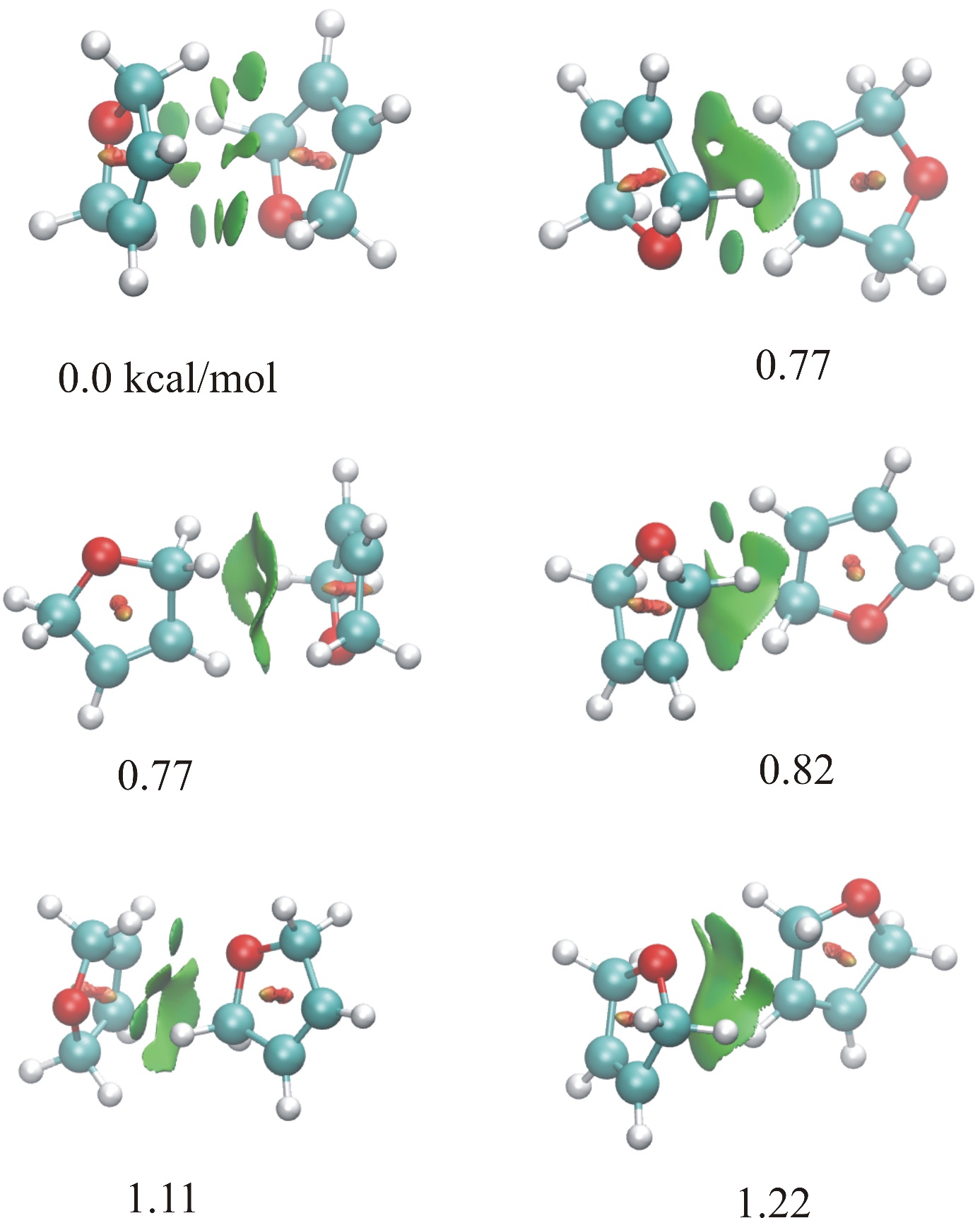


Fig. S11. NCI surfaces for 2,5-dihydrofuran dimers.


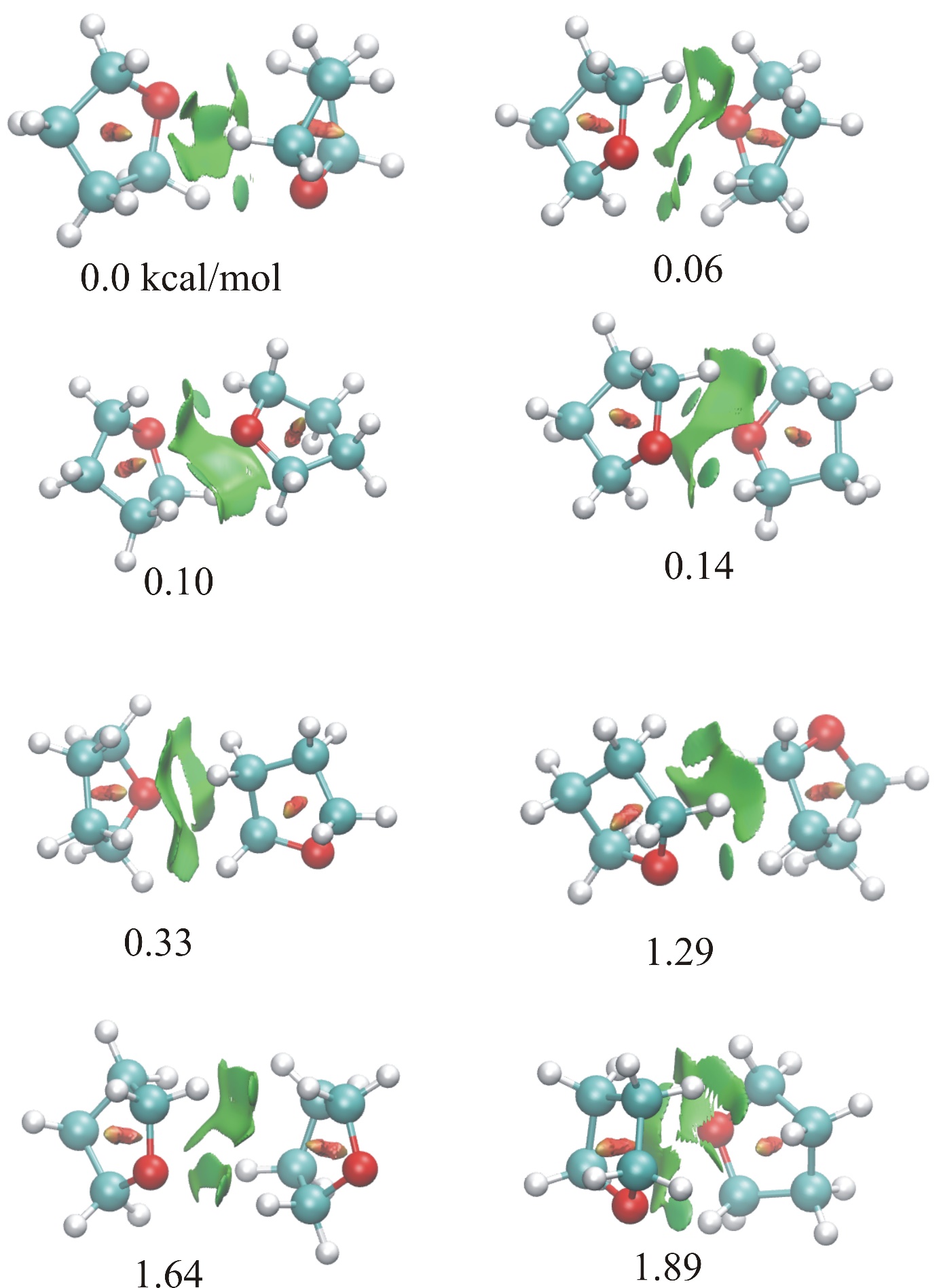


Fig. S12. NCI surfaces for tetrahydrofuran dimers.
